# Effect of Subcutaneous Injection of Tranexamic Acid on Ecchymosis and Edema After Oculofacial Surgery: A Prospective, Randomized, Split-Face, Double-Blind Study

**DOI:** 10.1093/asj/sjaf036

**Published:** 2025-03-11

**Authors:** Teresa H Chen, Dylann Fujimoto, Eduardo Damous Feijó, Jose Eduardo Rios, Marisa Novaes de Figueiredo Rassi, Rafael Leão, Jeremiah P Tao, Roberto Murillo Limongi

## Abstract

**Background:**

Tranexamic acid (TXA) is an antifibrinolytic that is regularly used to reduce bleeding in surgical specialties.

**Objectives:**

The objective of this study was to assess the effects of subcutaneous TXA in oculofacial plastic surgeries, with the hypothesis that TXA reduced postoperative ecchymosis and edema.

**Methods:**

This was a prospective, randomized, double-blind, split-face study. The sides of the face were randomized to local anesthetic (bupivacaine with epinephrine) mixed with TXA or sodium chloride (placebo). Photographs were taken immediately postoperatively and on postoperative day (POD) 7. Photographs were graded by 2 masked investigators with the Surgeon Periorbital Rating of Edema and Ecchymosis criteria. Patients selected the side that they subjectively determined to have less ecchymosis and edema. As a secondary outcome, patients rated pain on each side of their face with the Wong-Baker FACES pain scale.

**Results:**

Twenty-four patients undergoing bilateral, symmetric oculofacial surgery were included in the study. There was a statistically significant difference in postoperative periocular ecchymosis on POD7 (with TXA .91 ± 0.73 vs placebo 1.61 ± 1.03; *P* = .020) and in periocular edema on POD1 (with TXA 1.30 ± 0.76 vs placebo 2.00 ± 0.85; *P* = .028). All patients selected the side of the face receiving TXA as having less periocular ecchymosis and edema. There was no statistically significant difference in subjective pain level between the side receiving TXA vs placebo. There were no intraoperative or postoperative complications.

**Conclusions:**

Subcutaneous TXA was safe and reduced periocular ecchymosis and edema compared to contralateral placebo injections in this series of patients undergoing bilateral oculofacial plastic surgeries.

**Level of Evidence: 2:**

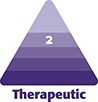

Tranexamic acid (TXA) is a synthetic lysine derivative that acts as an antifibrinolytic by blocking the binding site of lysine to plasminogen and preventing the conversion to plasmin, which normally lyses polymerized fibrin in blood clots.^1^ TXA is FDA approved for heavy menstrual bleeding and short-term prevention in patients with hemophilia, but is currently regularly used intravenously to reduce blood loss in a variety of surgical settings, including trauma, orthopedics, transplants, neurosurgery, and obstetrics. TXA has been shown to reduce all-cause mortality and risk of death from bleeding in trauma patients, reduce the need for transfusions in orthopedic, cardiac, and liver transplant surgeries, reduce the risk of rebleeding after a subarachnoid hemorrhage, and reduce blood loss after vaginal delivery and cesarean section when given prophylactically.^2-8^ In ophthalmology, it has been investigated for prevention of secondary hemorrhage after traumatic hyphema.^9^

TXA appears to be very safe and has not been associated with high risk of thromboembolic events. However, there have been reports of seizures when given in high doses systemically, and TXA was found to be an independent risk factor for seizures in larger studies.^5,6,10-12^ There have not been reports of adverse events such as seizures or thromboembolic events with local administration of tranexamic acid.

Reducing postoperative ecchymosis and edema after oculofacial plastic surgery procedures is important to minimize time away from work and activities for patients. Moreover, many inversely correlate the amount of bruising and swelling with the quality of the surgery. Importantly, more serious forms of ecchymosis, such as orbital compartment syndrome, predispose to neurologic injury.

Although several studies have shown reduced intraoperative bleeding, postoperative edema, and ecchymosis in rhinoplasties, the use of TXA in oculofacial plastic surgery has been minimally explored quantitatively and the results are, to date, equivocal.^13-17^ Sagiv et al and Chaichumporn et al showed no difference in postoperative edema and ecchymosis between the treatment group and controls for upper blepharoplasty.^18,19^ Marous et al report that in upper blepharoplasty intravenous TXA significantly reduced ecchymosis and edema scores compared to other patients randomized to placebo, and subcutaneous TXA reduced edema but not ecchymosis statistically significantly.^20^ Paramo et al showed that there was statistically significant reduction in ecchymosis scores in external levator advancement with subcutaneous TXA compared to contralateral placebo, but did they not reach statistically significant findings for upper lid blepharoplasty, combined upper and lower lid blepharoplasty, or conjunctival mullerectomy. There were no reported complications or adverse events with TXA in these studies.

The initial evidence for the benefit of locally injected TXA in ecchymosis and edema combined with its high safety profile makes its use intriguing. This study tested the hypothesis that subcutaneous TXA reduces both ecchymosis and edema in oculofacial surgeries.

## METHODS

This was a single-institution, double-blind, prospective, randomized, split-face clinical study. This study was approved, with formal ethical standards review by the Ethics Committee of the Universidade Federal de Goiás, Brazil, where patients of 4 affiliated oculofacial plastic surgeons were recruited. The collection and evaluation of protected patient health information was compliant with federal and local regulations and adhered to the ethical principles outlined in the Declaration of Helsinki as amended in 2013. Oral and written informed consent was obtained from the study participants. All clinical photographs were obtained with signed consent.

Inclusion criteria included patients over the age of 18 undergoing bilateral, symmetric oculofacial plastic procedures involving the eyelids between November 2023 and March 2024. The type of surgery and complications (if any) were recorded.

Exclusion criteria included pregnancy or breastfeeding, previous oculofacial plastic procedures, known allergy to TXA, personal or family history of clotting disorder, or personal history of thromboembolic events. All patients disclosed information about anticoagulant use and smoking status. Blood pressure was measured preoperatively and recorded. Sample size and power for statistical significance were calculated before data collection; it was determined that at least 16 patients or 32 sides needed to be recruited for a power of 80% and alpha of 0.05.

The 2 mixtures of subcutaneous local anesthesia were (1) 0.5% bupivacaine with 1:200,000 epinephrine and TXA 50 mg/mL in a 4:1 ratio for the TXA side and (2) 0.5% bupivacaine with 1:200,000 epinephrine and 0.9% sodium chloride in a 4:1 ratio for the placebo side. The side receiving TXA was randomized by flipping a coin, and the mixtures were prepared in advance by the anesthesiologist. Each side was injected with the local anesthesia mixture 10 minutes before incision to minimize positive or negative effect of time to injection while the contralateral procedure was performed. In the case of simultaneous surgery, both sides were injected with the local anesthesia mixture at the same time. The surgeon, patient, and graders were masked to which side received TXA vs saline. Grader 1 was a general ophthalmologist with 3 years of training and Grader 2 was an ASOPRS-trained oculofacial surgeon with 6 years of training.

Patient demographics, type of surgery, use of anticoagulant or antiplatelet medications, blood pressure, complications, and adverse events were recorded. Standardized, symmetric lighting and exposure, frontal photographs of each patient were taken immediately postoperatively and on postoperative days (PODs) 1 and 7.

The Surgeon Periorbital Rating of Edema and Ecchymosis (SPREE) criteria were used to grade periocular ecchymosis and edema. For periocular ecchymosis, 0 = no ecchymosis, 1 = ecchymosis up to one-third of lower and/or upper eyelids, 2 = ecchymosis up to two-thirds of lower and/or upper eyelids, and 3 = ecchymosis more than two-thirds of lower and/or upper eyelids. For periocular edema, 0 = no edema, 1 = no coverage of iris with eyelids, 2 = slight coverage of iris with eyelids, 3 = full coverage of iris with eyelids, and 4 = full closure of the eye.

The patients were interviewed on level of pain with the Wong-Baker FACES pain scale from 0 to 10 immediately postoperatively and on PODs 1 and 7 for each side of the face. The patients were also asked which side subjectively had less periocular ecchymosis and edema.

The kappa measure of agreement was applied to evaluate the scores of the 2 masked investigators, which were 0.63 (substantial) for periorbital ecchymosis and 0.72 (substantial) for periorbital edema, respectively. The chi-squared test was administered to compare degree of postoperative periocular ecchymosis, periocular edema, and subjective pain level between the side with and side without TXA. The statistical analysis was carried out with SPSS Statistics (IBM Corp., Armonk, NY).

## RESULTS

**Figure 1.**
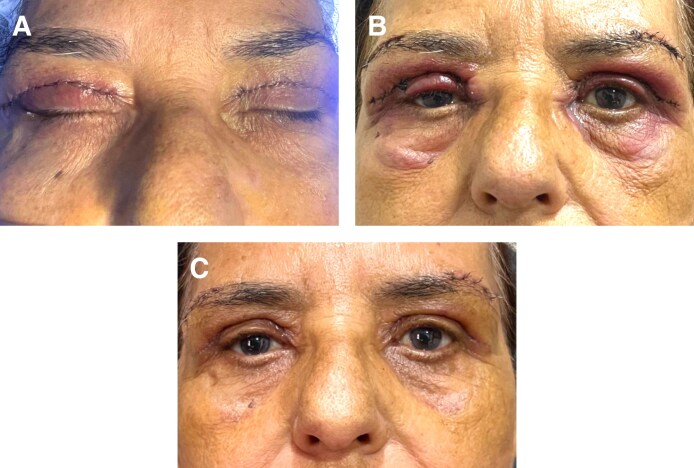
Postoperative photographs of a 59-year-old woman who underwent bilateral upper lid blepharoplasty and brow lift with administration of local anesthesia with (left side) and without (right side) tranexamic acid (A) immediately postoperatively, (B) on postoperative day 1, and (C) on postoperative day 7.

**Figure 2.**
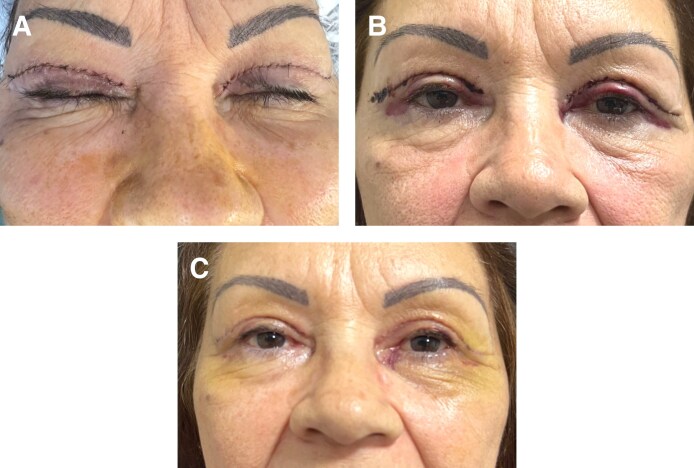
Postoperative photographs of a 53-year-old woman who underwent bilateral upper lid blepharoplasty with brow lift and conjunctival mullerectomy with local anesthesia with (right side) and without (left side) tranexamic acid (A) immediately postoperatively, (B) on postoperative day 1, and (C) on postoperative day 7.

**Figure 3.**
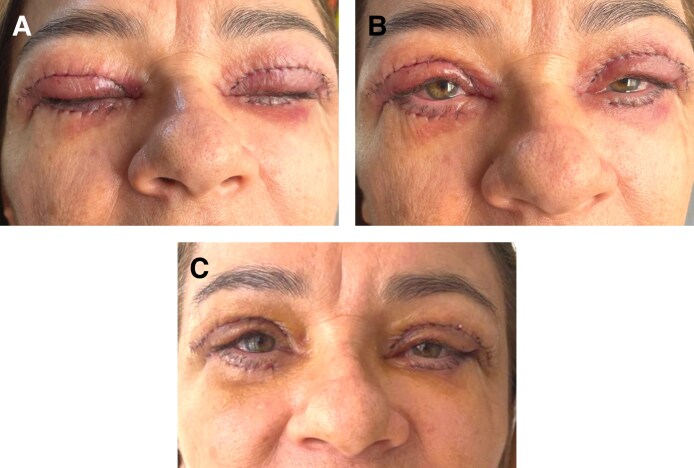
Postoperative photographs of a 59-year-old woman who underwent bilateral upper lid blepharoplasty, bilateral lower lid blepharoplasty, and midface lift with administration of local anesthesia with (right side) and without (left side) tranexamic acid (A) immediately postoperatively, (B) on postoperative day 1, and (C) on postoperative day 7.

Twenty-four patients were included. The demographics of the study patients are summarized in Table 1 and example photographs are shown in Figures 1-3. The average age was 58.0 ± 9.7 years old. The majority were women (22/24; 92%). Follow-up time was on average 10 months ± 3 months (range 3 months–12 months). Various oculofacial plastic surgeries involving the eyelids were performed, including bilateral upper lid blepharoplasty (BULB) with CO2 laser for incision (6; 25%), bilateral lower lid blepharoplasty (BLLB) with CO2 laser for incision (3; 13%), BULB, BLLB, and midface lift (11; 46%), BULB, BLLB, and midface lift with CO2 laser for incision (1; 4%), BULB and brow lift with CO2 laser for incision (2; 8%), and BULB, brow lift, and conjunctival mullerectomy with CO2 laser for incision (1; 4%). None of the patients were on anticoagulants or smoked. The average preoperative blood pressure (BP) was normotensive, with a systolic BP of 138 ± 13, diastolic BP of 73 ± 10, and mean arterial pressure of 95 ± 9. For the sides assigned to TXA, 13 (54%) were right side and 11 (46%) were left side.

**Table 1.** Baseline Patient Characteristics

Patient characteristics	
Age	58.0 ± 9.7
Sex	
Female	22 (92%)
Male	2 (8%)
Type of oculofacial plastic procedure	
Bilateral upper lid blepharoplasty (BULB) + CO2 laser	6 (25%)
Bilateral lower lid blepharoplasty (BLLB) + CO2 laser	3 (13%)
BULB + BLLB + midface lift	11 (46%)
BULB + BLLB + midface lift + CO2 laser	1 (4%)
BULB + brow lift + CO2 laser	2 (8%)
BULB + brow lift + conjunctival mullerectomy + CO2 laser	1 (4%)
History of anticoagulation use	
No	24 (100%)
Yes	0 (0%)
Smoking status	
No	24 (100%)
Yes	0 (0%)
Preoperative blood pressure (BP)	
Systolic BP	138 ± 13
Diastolic BP	73 ± 10
Mean arterial BP	95 ± 9
Side with tranexamic acid (TXA)	
Right	13 (54%)
Left	11 (46%)

There was a statistical significance in periocular ecchymosis between the 2 sides on POD 7 (with TXA 0.91 ± 0.73 vs placebo 1.61 ± 1.03; *P* = .020). The mean periocular ecchymosis score was less on the side of the face receiving TXA immediately postoperatively (with TXA 1.13 ± 0.74 vs placebo 1.67 ± 1.01; *P* = .13) and on POD 1 (with TXA 1.25 ± 0.74 vs placebo 1.63 ± 0.82; *P* = .35), but these did not reach statistical significance (Table 2).

**Table 2.** Postoperative Data: Side With and Side Without Tranexamic Acid

	Side with TXA	Side without TXA	*P* value
Periocular ecchymosis (SPREE, 0-3)			
Immediately postoperative	1.13 ± 0.74	1.67 ± 1.01	.13
POD1	1.25 ± 0.74	1.63 ± 0.82	.35
POD7	0.91 ± 0.73	1.61 ± 1.03	.020
Periocular edema (SPREE, 0-4)			
Immediately postoperative	1.33 ± 0.76	1.71 ± 0.86	.21
POD1	1.30 ± 0.76	2.00 ± 0.85	.028
POD7	0.83 ± 0.65	1.39 ± 0.89	.14
Patient subjective pain score (Wong-Baker FACES Pain Rating Scale, 0-10)			
Immediately postoperative	0.54 ± 0.93	0.54 ± 0.98	.84
POD1	0.29 ± 0.46	0.29 ± 0.46	1
POD7	0.18 ± 0.39	0.18 ± 0.39	1
Patient subjective better side	24 (100%)	0 (0%)	

POD, postoperative day; SPREE, Surgeon Periorbital Rating of Edema and Ecchymosis; TXA, tranexamic acid.

There was statistical significance in mean periocular edema score between the 2 sides on POD 1 (1.30 ± 0.76 vs placebo 2.00 ± 0.85; *P* = .028). Mean edema score was less on the side of the face receiving TXA immediately postoperatively (with TXA 1.33 ± 0.76 vs placebo 1.71 ± 0.86; *P* = .21) and on POD 7 (with TXA 0.83 ± 0.65 vs placebo 1.39 ± 0.89; *P* = .14), but these were not statistically significant (Table 2).

All patients, masked to the intervention, selected the side receiving TXA as subjectively having less ecchymosis and edema. No intraoperative or postoperative complications or adverse events occurred (Table 2).

There was no statistically significant difference in pain level between the side with TXA and the side with placebo (Table 2).

## DISCUSSION

In this study, injection of TXA was associated with a reduction in mean ecchymosis and edema scores at all time points; statistically significant findings were on POD 7 for ecchymosis and POD 1 for edema. There were no reported complications or adverse effects, and subjective metrics corroborated the benefit of TXA in reducing ecchymosis and edema. In fact, all patients, although blinded to the intervention, selected the side of the face receiving TXA as the side with less bruising and swelling. Pain scores were not different between the sides of the face.

These findings are consistent with previous literature on facial plastic surgeries, in which TXA significantly decreased ecchymosis and edema in patients undergoing rhinoplasties and facelift surgeries.^14,16,17,21^ Our findings differ from those of Sagiv et al, Paramo et al, and Chaichumporn et al, who found that subcutaneous TXA did not significantly reduce ecchymosis compared to placebo in upper lid blepharoplasty.^18,19,22^ Sagiv et al acknowledge that their study was underpowered. The data of Paramo et al for blepharoplasty trended toward significance; their overall data set may have been limited by the inclusion of mostly (92%) upper eyelid surgeries. Paramo et al, however, demonstrated statistically significant reduction in ecchymosis in levator advancement with subcutaneous TXA compared to placebo in their split-face study. Interestingly, Chaichumporn et al did not find a statistically significant reduction in either ecchymosis or edema and actually found a higher intraoperative blood loss in the TXA group.^19^ Similar to Marous et al, our study indicated that TXA may reduce postoperative edema, with statistically significant findings on POD1.^20^

One key difference between our study and previous studies was the TXA concentration. In our study TXA was 50 mg/mL, compared to 100 mg/mL in other studies. Moreover, our admixture was 4:1 local to TXA (50 mg/mL) yielding a TXA concentration of 10 mg/mL. Both the Sagiv et al and Marous et al studies administered a higher TXA concentration of 1:1 local to TXA (100 mg/mL), with final TXA concentration of 50 mg/mL. Chaichumporn et al gave a lower TXA concentration of 1:1 local to TXA (50 mg/mL), with final TXA concentration of 25 mg/mL. Paramo et al used a similar lower TXA concentration of 9:1 local to TXA (100 mg/mL), with final TXA concentration of 10 mg/mL. Pain levels reported by this study's patients were almost identical between the side with TXA and the side with placebo, corroborating previous studies that TXA did not change the anesthetic effect of local anesthesia through dilution, change in pH, or other mechanisms.^18,23^

A limitation of this study, like other split-face studies, was the potential of diffuse effects and crossover to the side of the face not receiving the pharmacologic intervention. Nevertheless, the study design was able to detect statistically significant differences between the intervention vs placebo sides. Another limitation of this study was that intraoperative blood loss between the 2 sides was not assessed, and TXA's benefits in reducing serious hemorrhagic complications were not borne out by the data. TXA has been shown to decrease blood loss and the need for transfusions in high blood loss surgeries, therefore there are theoretically similar benefits in oculofacial plastic surgeries. A meta-analysis of TXA use in aesthetic surgery patients by Laikhter et al showed an average blood loss reduction of 26.3 mL, with a trend toward decreased odds of postoperative hematoma formation.^24^ The decrease in blood loss with a resultant dry surgical field has also been shown to significantly reduce operating time and drain output in facelifts.^25-27^ However, because hemorrhagic complications are rare (0.055%) in eyelid surgeries, detecting for these benefits was beyond the scope of our study.^28^ In the umbrella review by Ahmed et al, which included rhinoplasties, septorhinoplasties, rhytidectomies, and blepharoplasties, the data showed that TXA reduced blood loss volume and duration of surgery in all surgeries except for blepharoplasties, likely due to the minimal blood loss encountered in oculofacial surgeries.^29^ The effects of TXA in reducing risk of orbital compartment syndrome and other hemorrhagic events therefore remains uncertain. Finally, although the study was powered to detect a difference between the TXA and non-TXA sides, the number of participants remained relatively small, and diverse oculofacial procedures were included. A larger-scale study would provide subgroup analysis based on procedures and better detect rare complications and adverse events from subcutaneous TXA administration. It would be of interest as well to analyze ecchymosis and edema at a longer follow-up, after 1 week. However, in the authors' practice, after the initial first week postoperatively, the next scheduled follow-up is at 3 months, and patients do not have residual ecchymosis or edema by that time.

The implications of the data presented in this study are that TXA injections may be associated with a clinically meaningful reduction of ecchymosis and edema after oculofacial plastic surgery. Given that TXA injections are inexpensive (around 48 US dollars per 100 mL) and appear to be very safe, there may be a low barrier for oculofacial and facial plastic surgeons to implement them in practice. Wokes et al demonstrated through survey research of 93 British Association of Aesthetic Plastic Surgeons (BAAPS) members that a majority (78%) utilizes TXA regularly in aesthetic procedures, including periorbital surgeries such as blepharoplasties, face and neck lifts, and brow lifts, with reduced intraoperative and postoperative bleeding and ecchymosis cited as the main benefits.^15^

## CONCLUSIONS

In conclusion, subcutaneous tranexamic acid reduced postoperative periocular ecchymosis and edema and had no effect on subjective pain level in this split-face study. Further studies are needed to test whether there is a dose-dependent effect and to assess for other effects of the use of TXA in oculofacial plastic surgeries.
